# Galectin-9, FABP4 and pentraxin-3 are associated with myocardial dysfunction and discriminate chemotherapy-related cardiac dysfunction in anthracycline-treated breast cancer: a single-centre retrospective study

**DOI:** 10.3389/fonc.2026.1913970

**Published:** 2026-08-26

**Authors:** Jincheng Ma, Zhiqiang Ma, Yanhui Cheng, Xiaoyun Li, Huiqing Song, Shuai Wang

**Affiliations:** 1 Department of Oncology, The First Affiliated Hospital of Henan University, Kaifeng, Henan, China; 2 Department of Breast Surgery, The First Affiliated Hospital of Henan University, Kaifeng, Henan, China

**Keywords:** anthracycline, biomarker discrimination, breast cancer, cancer therapy-related cardiac dysfunction, galectin-9, global longitudinal strain, pentraxin-3

## Abstract

**Background:**

Anthracycline-based chemotherapy improves breast cancer outcomes but may cause cancer therapy-related cardiac dysfunction (CTRCD). Biomarkers reflecting inflammatory and metabolic myocardial stress may complement imaging surveillance, but the combined value of galectin-9 (Gal-9), fatty-acid-binding protein 4 (FABP4) and pentraxin-3 (PTX-3) is unclear.

**Objective:**

This study aimed to evaluate whether serum Gal-9, FABP4 and PTX-3 are associated with myocardial dysfunction and discriminate CTRCD.

**Methods:**

This single-centre retrospective cohort included 219 women receiving at least three anthracycline-based cycles from December 2022 to June 2025. Patients were classified as CTRCD (n = 66) or non-CTRCD (n = 153) using ASE/EACVI and ESC criteria. Baseline and post-cycle-3 echocardiography and hs-cTnI were recorded; post-cycle-3 biomarkers were measured by ELISA. Analyses included adjusted correlations, multivariable logistic regression, ROC/DeLong testing and bootstrap validation.

**Results:**

Baseline characteristics were comparable. After chemotherapy, Gal-9, FABP4 and PTX-3 were higher, whereas LVEF, |GLS| and MCI were lower, in the CTRCD group (all P < 0.05). Biomarkers correlated inversely with LVEF, |GLS| and MCI after adjustment (all P < 0.001). The combined biomarker score was independently associated with CTRCD (adjusted OR 3.25 per SD; 95% CI 2.09–5.07; P < 0.001). The combined model achieved an AUC of 0.880 (95% CI 0.829–0.920), optimism-corrected AUC of 0.854 and cross-validated AUC of 0.846, improving the clinical-model AUC from 0.642 to 0.872 (P < 0.001).

**Conclusion:**

Gal-9, FABP4 and PTX-3 were associated with concurrent myocardial dysfunction and improved discrimination of on-treatment CTRCD. This candidate adjunctive panel requires serial sampling and external validation before clinical use.

## 1 Introduction

Breast cancer remains the most frequently diagnosed malignancy in women worldwide and represents a major source of long-term morbidity despite substantial advances in screening, systemic therapy, surgery and radiotherapy (1). As survival has improved, the clinical priorities of breast cancer care have expanded beyond tumour control to include treatment-related complications that affect quality of life, functional status and long-term survival. Among these complications, cardiovascular disease has become increasingly important. In breast cancer survivors, cardiovascular morbidity and mortality may rival, and in some subgroups exceed, cancer recurrence as a determinant of late outcome, particularly in patients exposed to potentially cardiotoxic systemic therapy (2, 3). This overlap between oncological benefit and cardiovascular risk has made cardio-oncology surveillance an essential component of contemporary breast cancer management.

Anthracyclines remain a key component of treatment for many patients with breast cancer, but their clinical utility is limited by cumulative and sometimes irreversible myocardial injury. Anthracycline-associated cardiotoxicity is mediated through several interacting mechanisms, including topoisomerase-IIβ–related DNA damage, oxidative stress, mitochondrial dysfunction, impaired energetic homeostasis and progressive cardiomyocyte loss (3, 4). In patients with human epidermal growth factor receptor 2 (HER2)-positive disease, trastuzumab may further increase cardiac vulnerability because HER2 signalling contributes to cardiomyocyte survival and adaptive stress responses (5). The central clinical challenge is therefore to identify myocardial injury at a stage when intervention, treatment modification or intensified surveillance may still prevent progression to clinically overt heart failure, without compromising the antitumour efficacy of chemotherapy (2, 6).

Current definitions of cancer therapy-related cardiac dysfunction (CTRCD) rely on a combination of imaging findings, functional change and circulating cardiac biomarkers. The 2014 ASE/EACVI consensus and the 2022 ESC cardio-oncology guideline emphasise changes in left ventricular ejection fraction (LVEF), relative reduction in global longitudinal strain (GLS), and elevation of cardiac troponin or natriuretic peptides as key indicators of treatment-related myocardial injury (6, 7). LVEF remains widely used because it is familiar, clinically interpretable and strongly linked to heart failure outcomes. However, it is relatively insensitive to early subclinical injury and may decline only after substantial myocardial reserve has already been lost. GLS provides a more sensitive measure of longitudinal systolic deformation and can identify early impairment even when LVEF is preserved (7, 8). Nevertheless, imaging-based assessment alone does not fully capture the biological pathways that precede or accompany myocardial dysfunction.

Circulating biomarkers may complement echocardiographic surveillance because they are minimally invasive, repeatable and capable of reflecting molecular processes that are not directly visible on routine imaging. Troponin and natriuretic peptides are already incorporated into cardio-oncology practice, but interest is increasing in additional biomarkers that reflect inflammation, metabolic stress, endothelial activation, extracellular matrix remodelling and fibrosis (9). Galectin-9 (Gal-9) is a β-galactoside-binding lectin involved in immune-inflammatory signalling and tissue remodelling, and has been implicated in pathways relevant to myocardial inflammation and fibrosis (10). Fatty-acid-binding protein 4 (FABP4) links adipose-tissue metabolism, lipid handling and inflammatory activation, and may reflect metabolic stress that contributes to impaired cardiomyocyte energetics (9). Pentraxin-3 (PTX-3), a long pentraxin acute-phase protein, is released locally at sites of vascular and myocardial injury and has been associated with adverse cardiovascular outcomes (11). Together, these three biomarkers represent biologically distinct but potentially complementary axes of myocardial stress: immune-inflammatory activation, lipid-metabolic dysfunction and acute-phase vascular injury.

Despite this biological rationale, the clinical relevance of Gal-9, FABP4 and PTX-3 in anthracycline-treated breast cancer remains insufficiently defined. Previous studies have evaluated several circulating markers in chemotherapy-associated cardiotoxicity, but the combined behaviour of these three biomarkers, their relationship with echocardiographic functional indices, and their incremental discriminatory value beyond standard clinical information are not well established (9–11). This gap is clinically important because CTRCD is heterogeneous: some patients develop measurable functional decline, whereas others show biomarker elevation or strain abnormalities before overt reduction in LVEF. A biomarker panel reflecting multiple injury pathways may therefore provide a more integrated picture of on-treatment myocardial stress than any single analyte. However, because retrospective observational data cannot establish temporal precedence or causality, such markers should first be evaluated cautiously as associative and discriminatory candidates requiring prospective validation.

Therefore, this study aimed to evaluate, in women with breast cancer receiving anthracycline-based chemotherapy, whether serum Gal-9, FABP4 and PTX-3 differ between patients with and without on-treatment CTRCD; whether these biomarkers are associated with echocardiographic indices of myocardial function, including LVEF, GLS and myocardial composite index, before and after adjustment for relevant clinical confounders; and whether the three biomarkers, individually and in combination, discriminate CTRCD with internally validated performance and incremental value beyond standard clinical information.

## 2 Methods

### 2.1 Study design and participants

This single-centre retrospective cohort study was conducted in the Department of Oncology, The First Affiliated Hospital of Henan University, and was reported in accordance with the Strengthening the Reporting of Observational Studies in Epidemiology (STROBE) recommendations. The study protocol and the use of de-identified clinical data were approved by the Ethics Committee of The First Affiliated Hospital of Henan University (approval number SKh-202507JY). Consecutive women with histologically confirmed breast cancer who received anthracycline-based chemotherapy between December 2022 and June 2025 were screened for eligibility. All patients received an epirubicin- or doxorubicin-based regimen administered according to body surface area on a 21-day cycle for at least three consecutive cycles. Patients with human epidermal growth factor receptor 2 (HER2)-positive disease additionally received trastuzumab, administered sequentially after anthracycline exposure at a loading dose of 8 mg/kg followed by 6 mg/kg maintenance dosing.

**Figure 1:**
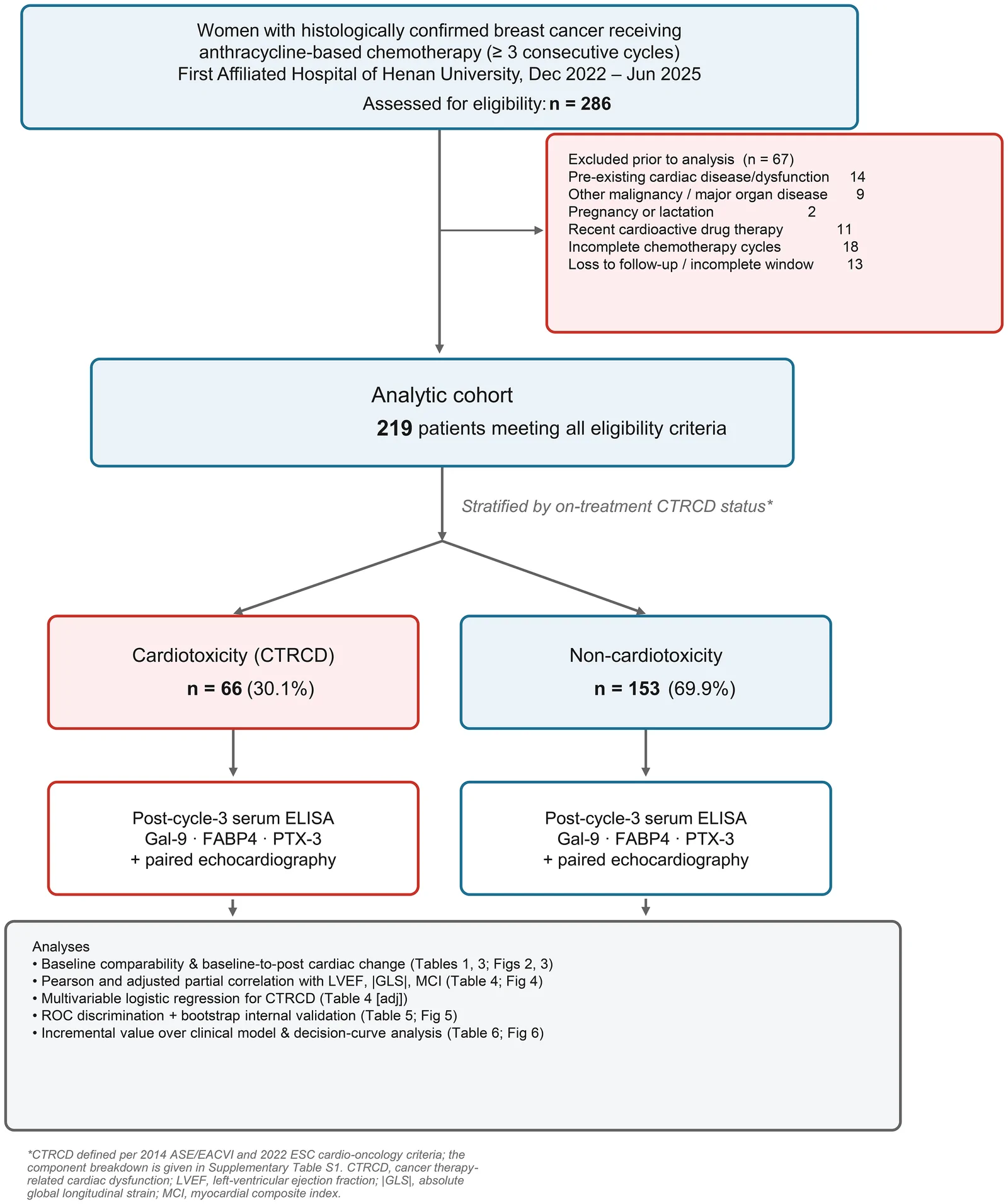
Cohort flow (STROBE). Of 286 women screened, 67 were excluded for the reasons shown, leaving 219 in the analytic cohort, stratified by on-treatment cancer therapy-related cardiac dysfunction (CTRCD) into cardiotoxicity (n = 66) and non-cardiotoxicity (n = 153) groups. CTRCD was defined per the 2014 ASE/EACVI consensus and 2022 ESC cardio-oncology guideline; the component breakdown is in **Supplementary Table 1**.

Patients were eligible if they had a pathological diagnosis of breast cancer according to the China Anti-Cancer Association guidelines (12), completed at least three consecutive standard chemotherapy cycles, and had available baseline and post-treatment serum biomarker and echocardiographic data. Patients were excluded if they had documented structural heart disease or cardiac dysfunction before chemotherapy, a concurrent second malignancy or severe organic disease, pregnancy or lactation, exposure within the preceding three months to drugs likely to affect cardiac function, incomplete chemotherapy cycles, or an incomplete biomarker or echocardiographic assessment window. Among 286 women screened, 67 were excluded, leaving 219 patients in the analytic cohort (**Figure 1**). No patient was excluded because of a treatment modification prompted by a cardiac adverse event; the small number of regimen modifications during the study period were attributable to non-cardiac toxicities.

### 2.2 Outcome definition and group allocation

The primary outcome was on-treatment cancer therapy-related cardiac dysfunction (CTRCD), adjudicated according to the 2014 ASE/EACVI multimodality-imaging consensus and the 2022 ESC cardio-oncology guideline (6, 7). CTRCD was defined as the presence of at least one of the following during treatment: a reduction in left ventricular ejection fraction (LVEF) of more than 10 absolute percentage points from baseline to a value below 53%; a new relative reduction in absolute global longitudinal strain (|GLS|) of more than 15% from baseline; elevation of high-sensitivity cardiac troponin I (hs-cTnI) above the 99th-percentile upper reference limit; new symptomatic heart failure; or an arrhythmia requiring clinical intervention.

Patients meeting any CTRCD criterion were assigned to the cardiotoxicity group (n = 66), whereas those who did not meet any criterion were assigned to the non-cardiotoxicity group (n = 153). Because diagnostic criteria could overlap, the contribution of each criterion and the mutually exclusive primary classification pathway are provided in **Supplementary Table 1**. Sensitivity analyses were pre-specified to assess the robustness of the findings after restricting the outcome to imaging-supported CTRCD and after excluding patients classified by hs-cTnI elevation alone, as described in Section 2.5. Because hs-cTnI forms part of this composite outcome and is itself a circulating marker, these analyses were planned specifically to confirm that the discriminatory value of the candidate panel did not depend on the troponin component of the endpoint. The three analytes evaluated here, galectin-9, FABP4 and pentraxin-3, are biochemically and mechanistically distinct from cardiac troponin and did not contribute to case ascertainment. Biomarker measurement and echocardiographic acquisition and interpretation were performed by clinicians and laboratory personnel blinded to group allocation.

### 2.3 Serum biomarker measurement

Within 24 h after completion of the third chemotherapy cycle, fasting morning venous blood samples of 5 mL were collected and centrifuged at 3500 r/min for 10 min. Serum was separated promptly, stored at −80 °C, and assayed in a single batch to minimise inter-assay variation. Serum galectin-9 (Gal-9), fatty-acid-binding protein 4 (FABP4) and pentraxin-3 (PTX-3) concentrations were quantified using enzyme-linked immunosorbent assay (ELISA) kits according to the manufacturers’ instructions. All assays were performed by operators blinded to clinical and echocardiographic data.

For the three assays, the intra-assay and inter-assay coefficients of variation were <10% and <15%, respectively. The lower limits of detection were <0.1 pg/mL for Gal-9, <31.25 pg/mL for FABP4 and <39.06 pg/mL for PTX-3. Full assay characteristics, including manufacturer, catalogue number, detection range, sample handling and dilution procedures, are provided in **Supplementary Table 2**.

### 2.4 Echocardiography

Transthoracic echocardiography was performed at baseline before chemotherapy and again within the same post-cycle-3 assessment window as biomarker sampling. Examinations were conducted using a Philips iU22 ultrasound system (Philips Healthcare, the Netherlands), with patients positioned in the left lateral decubitus position. LVEF was measured using the biplane Simpson method. Pulsed-wave Doppler and tissue Doppler imaging were used to derive early-to-late mitral inflow velocity ratio (E/A) and early mitral inflow velocity to early diastolic mitral annular velocity ratio (E/e′).

A matrix three-dimensional probe was used for speckle-tracking analysis of global radial strain (GRS), global longitudinal strain, reported as an absolute value (|GLS|), and a vendor-software-derived myocardial composite index (MCI). Because MCI is generated automatically by the analysis platform and is not yet standardised across vendors, it was treated as an exploratory echocardiographic index. Because vendor-specific deformation metrics of this kind are not interchangeable across ultrasound platforms, MCI was regarded as strictly exploratory, was used only to complement the validated indices of LVEF and |GLS|, and was not intended for cross-vendor comparison or for clinical decision-making. The timing of baseline and post-treatment imaging relative to chemotherapy is summarised in **Supplementary Table 3**. Intraobserver and interobserver reproducibility, assessed using intraclass correlation coefficients, is reported in **Supplementary Table 4**. All echocardiographic examinations were performed and independently interpreted by two senior sonographers blinded to clinical and biomarker data. Each parameter was measured at least three times and averaged. Imaging acquisition and interpretation followed ASE/EACVI recommendations (7).

### 2.5 Statistical analysis

Continuous variables were assessed for normality using the Shapiro–Wilk test. Normally distributed variables were summarised as mean ± standard deviation and compared using the independent-samples t-test, whereas non-normally distributed variables were summarised as median and interquartile range and compared using the Mann–Whitney U test. Categorical variables were summarised as number and percentage and compared using the χ² test. Baseline-to-post-treatment changes in cardiac measures were calculated and compared between groups.

Associations between serum biomarkers and echocardiographic indices were first evaluated using Pearson correlation coefficients. To account for potential confounding, partial correlations were then calculated after adjustment for age, body mass index, hypertension, diabetes, hyperlipidaemia, trastuzumab use, doxorubicin-equivalent anthracycline dose and baseline LVEF. These adjusted correlation analyses are reported in **Supplementary Table 5**.

Multivariable logistic regression was used to evaluate the association between serum biomarkers and CTRCD. Clinically relevant covariates were selected *a priori* and included age, body mass index, hypertension, diabetes, hyperlipidaemia, trastuzumab use, doxorubicin-equivalent anthracycline dose and baseline LVEF. Biomarkers were standardised before modelling and expressed per 1-standard-deviation increase. Gal-9, FABP4 and PTX-3 were entered either simultaneously as individual biomarkers or as a single combined logistic-regression-derived biomarker score. Because the number of CTRCD events was limited, multivariable models were kept parsimonious to reduce the risk of model overfitting.

The discriminatory performance of each biomarker and the combined biomarker model for CTRCD was evaluated using receiver-operating-characteristic (ROC) analysis. The optimal cut-off was determined using the maximum Youden index, and areas under the ROC curve (AUCs) were compared using the DeLong test (13). Internal validation was performed using 1000-resample bootstrapping to estimate optimism-corrected AUC, calibration slope and Brier score, supplemented by repeated 10-fold cross-validation (14). Incremental value beyond a clinical model was assessed by change in AUC, integrated discrimination improvement and category-free net reclassification improvement (15). Clinical usefulness was further evaluated using decision-curve analysis (16).

Pre-specified sensitivity analyses repeated the combined-model discrimination after excluding trastuzumab-treated patients, restricting the analysis to the epirubicin-only subgroup, limiting the outcome to imaging-supported CTRCD defined by LVEF and/or |GLS| criteria, and excluding patients classified as CTRCD solely on the basis of hs-cTnI elevation. An additional sensitivity analysis adjusted the combined-panel model for tumour stage and tumour size to examine residual confounding by malignancy-related disease burden. Statistical analyses were performed using SPSS version 26.0 (IBM Corp., Armonk, NY, USA), MedCalc version 11.4 (MedCalc Software, Ostend, Belgium) and R version 4.3 (R Foundation for Statistical Computing). A two-sided P value <0.05 was considered statistically significant.

Because this was a retrospective study including all eligible patients treated during the study period, no formal prospective sample-size calculation was performed. The final analytic cohort included 66 patients with CTRCD and 153 without CTRCD, allowing estimation of a parsimonious three-biomarker model. Statistical precision was expressed using 95% confidence intervals around effect estimates and AUCs, and the potential for optimism was evaluated through bootstrap internal validation.

## 3 Results

### 3.1 Study population and patient flow

Between December 2022 and June 2025, 286 women with histologically confirmed breast cancer who received anthracycline-based chemotherapy were assessed for eligibility. Of these, 67 were excluded before analysis because of pre-existing cardiac disease or dysfunction (n = 14), other malignancy or major organ disease (n = 9), pregnancy or lactation (n = 2), recent cardioactive drug therapy (n = 11), incomplete chemotherapy cycles (n = 18), or loss to follow-up or an incomplete assessment window (n = 13). The final analytic cohort included 219 patients, of whom 66 met the criteria for on-treatment cancer therapy-related cardiac dysfunction (CTRCD) and 153 did not. The observed frequency of CTRCD was 30.1% (66/219; 95% CI, 24.4–36.5%). Patient flow and group allocation are shown in **Figure 1**.

### 3.2 Baseline demographic, clinical and treatment characteristics

Baseline characteristics were broadly comparable between the CTRCD and non-CTRCD groups (**Table 1**). Mean age was 48.31 ± 3.20 years in the CTRCD group and 47.89 ± 3.17 years in the non-CTRCD group (P = 0.371), and body mass index was similar between groups (20.73 ± 0.88 vs 20.59 ± 0.82 kg/m²; P = 0.258). The proportions of patients with hypertension, diabetes mellitus, hyperlipidaemia and current or former smoking did not differ significantly between groups. Baseline systolic blood pressure and estimated glomerular filtration rate were also similar.

**Table 1:** Baseline demographic, cardiac and treatment characteristics by cardiotoxicity status.

Characteristic	CTRCD (n = 66)	Non-CTRCD (n = 153)	*P* value
Demographics and cardiovascular risk			
Age, years	48.31 ± 3.20	47.89 ± 3.17	0.371
Body mass index, kg/m^2^	20.73 ± 0.88	20.59 ± 0.82	0.258
Hypertension	14 (21.2)	25 (16.3)	0.387
Diabetes mellitus	9 (13.6)	17 (11.1)	0.596
Hyperlipidaemia	12 (18.2)	21 (13.7)	0.398
Current/former smoking	6 (9.1)	11 (7.2)	0.626
Baseline systolic BP, mmHg	123.6 ± 11.5	122.1 ± 10.8	0.354
Baseline eGFR, mL/min/1.73 m^2^	93.4 ± 14.2	95.1 ± 13.5	0.402
Baseline cardiac status			
Baseline LVEF, %	67.11 ± 4.62	67.38 ± 4.35	0.679
Baseline |GLS|, %	20.23 ± 2.05	20.47 ± 2.01	0.421
Baseline hs-cTnI, ng/mL	0.008 ± 0.004	0.007 ± 0.003	0.071
Baseline NT-proBNP, pg/mL	71.2 ± 38.4	64.5 ± 34.7	0.198
Tumour and treatment			
Tumour stage III–IV	37 (56.1)	91 (59.5)	0.638
Ductal carcinoma	50 (75.8)	133 (86.9)	0.121
Tumour size, mm	12.27 ± 1.48	11.95 ± 1.39	0.127
HER2-positive disease	23 (34.8)	36 (23.5)	0.083
Trastuzumab use	23 (34.8)	36 (23.5)	0.083
Epirubicin-based regimen	52 (78.8)	128 (83.7)	0.389
Doxorubicin-based regimen	14 (21.2)	25 (16.3)	0.389
Doxorubicin-equivalent dose, mg/m^2^	242.6 ± 41.3	235.4 ± 38.7	0.218
Taxane-containing regimen	44 (66.7)	99 (64.7)	0.779
Left-sided radiotherapy	20 (30.3)	40 (26.1)	0.522
Endocrine therapy	35 (53.0)	77 (50.3)	0.713
Cardioprotective medication during chemotherapy			
ACEI/ARB	9 (13.6)	17 (11.1)	0.596
Beta-blocker	6 (9.1)	12 (7.8)	0.752
Statin	10 (15.2)	19 (12.4)	0.579
Dexrazoxane	8 (12.1)	18 (11.8)	0.941

Data are mean ± SD or n (%). ACEI, angiotensin-converting-enzyme inhibitor; ARB, angiotensin-receptor blocker; BP, blood pressure; CTRCD, cancer therapy-related cardiac dysfunction; eGFR, estimated glomerular filtration rate; GLS, global longitudinal strain (absolute value); HER2, human epidermal growth factor receptor 2; hs-cTnI, high-sensitivity cardiac troponin I; LVEF, left ventricular ejection fraction; NT-proBNP, N-terminal pro-B-type natriuretic peptide.

Baseline cardiac status was balanced between groups. Baseline LVEF was 67.11 ± 4.62% in the CTRCD group and 67.38 ± 4.35% in the non-CTRCD group (P = 0.679), while baseline |GLS| was 20.23 ± 2.05% and 20.47 ± 2.01%, respectively (P = 0.421). Baseline hs-cTnI and NT-proBNP values showed no statistically significant between-group differences. Tumour stage, pathological subtype, tumour size, HER2-positive disease, trastuzumab use, anthracycline regimen, doxorubicin-equivalent cumulative dose, taxane-containing therapy, left-sided radiotherapy and endocrine therapy were not significantly different between groups. Use of cardioprotective medications during chemotherapy, including ACEI/ARB, beta-blockers, statins and dexrazoxane, was also similar between groups.

### 3.3 Post-chemotherapy serum biomarkers and echocardiographic indices

**Figure 2:**
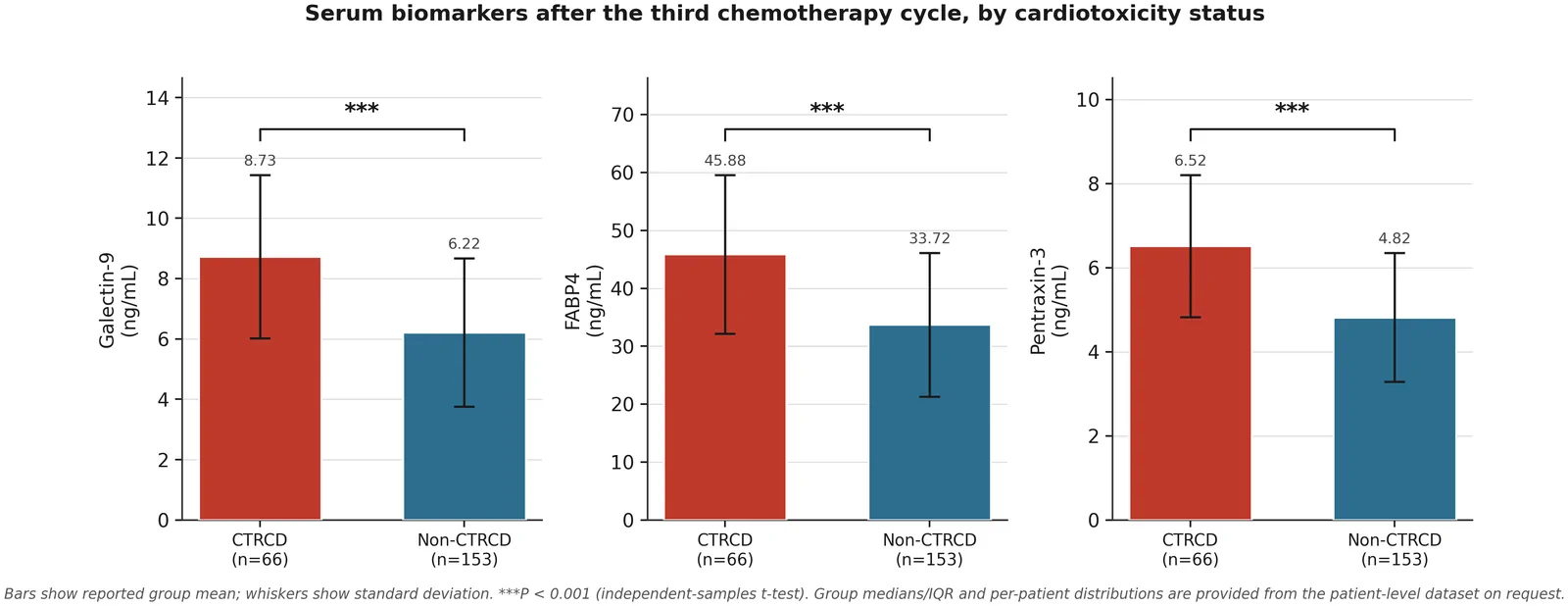
Serum biomarkers after the third chemotherapy cycle, by group. Bars show group mean and whiskers the standard deviation for galectin-9, FABP4 and pentraxin-3, all higher in the cardiotoxicity group (***P < 0.001; independent-samples t-test).

After the third chemotherapy cycle, serum concentrations of all three biomarkers were higher in the CTRCD group than in the non-CTRCD group (**Table 2**; **Figure 2**). Galectin-9 was 8.73 ± 2.71 ng/mL in the CTRCD group compared with 6.22 ± 2.46 ng/mL in the non-CTRCD group, corresponding to a mean difference of 2.51 ng/mL (95% CI, 1.74–3.28; P < 0.001). FABP4 was also higher in the CTRCD group (45.88 ± 13.72 vs 33.72 ± 12.40 ng/mL; mean difference, 12.16 ng/mL; 95% CI, 8.27–16.05; P < 0.001). PTX-3 showed a similar pattern (6.52 ± 1.69 vs 4.82 ± 1.53 ng/mL; mean difference, 1.70 ng/mL; 95% CI, 1.22–2.18; P < 0.001).

**Table 2:** Post-chemotherapy serum biomarkers and echocardiographic indices by group.

Variable	CTRCD (n = 66)	Non-CTRCD (n = 153)	*t*	*P*
Gal-9, ng/mL	8.73 ± 2.71	6.22 ± 2.46	6.717	< 0.001
FABP4, ng/mL	45.88 ± 13.72	33.72 ± 12.40	6.446	< 0.001
PTX-3, ng/mL	6.52 ± 1.69	4.82 ± 1.53	7.308	< 0.001
LVEF, %	63.88 ± 5.32	65.93 ± 6.29	2.314	0.022
E/e′	6.73 ± 0.82	6.48 ± 0.71	1.368	0.173
E/A	1.53 ± 0.29	1.48 ± 0.24	1.326	0.186
GRS, %	48.94 ± 6.84	48.59 ± 6.72	0.352	0.725
|GLS|, %	14.77 ± 2.39	17.59 ± 2.71	7.314	< 0.001
MCI, %	250.49 ± 41.93	299.74 ± 47.60	7.664	< 0.001

Data are mean ± SD. FABP4, fatty-acid-binding protein 4; Gal-9, galectin-9; GLS, global longitudinal strain (absolute value); GRS, global radial strain; LVEF, left ventricular ejection fraction; MCI, myocardial composite index; PTX-3, pentraxin-3.

Post-chemotherapy systolic-function indices were lower in the CTRCD group. LVEF was 63.88 ± 5.32% in the CTRCD group and 65.93 ± 6.29% in the non-CTRCD group, with a mean difference of −2.05 percentage points (95% CI, −3.69 to −0.41; P = 0.022). Absolute GLS was 14.77 ± 2.39% in the CTRCD group and 17.59 ± 2.71% in the non-CTRCD group (mean difference, −2.82 percentage points; 95% CI, −3.55 to −2.09; P < 0.001). MCI was also lower in the CTRCD group (250.49 ± 41.93% vs 299.74 ± 47.60%; mean difference, −49.25 percentage points; 95% CI, −61.98 to −36.52; P < 0.001). In contrast, E/e′, E/A and GRS did not differ significantly between groups.

### 3.4 Baseline-to-post-treatment cardiac changes

**Figure 3:**
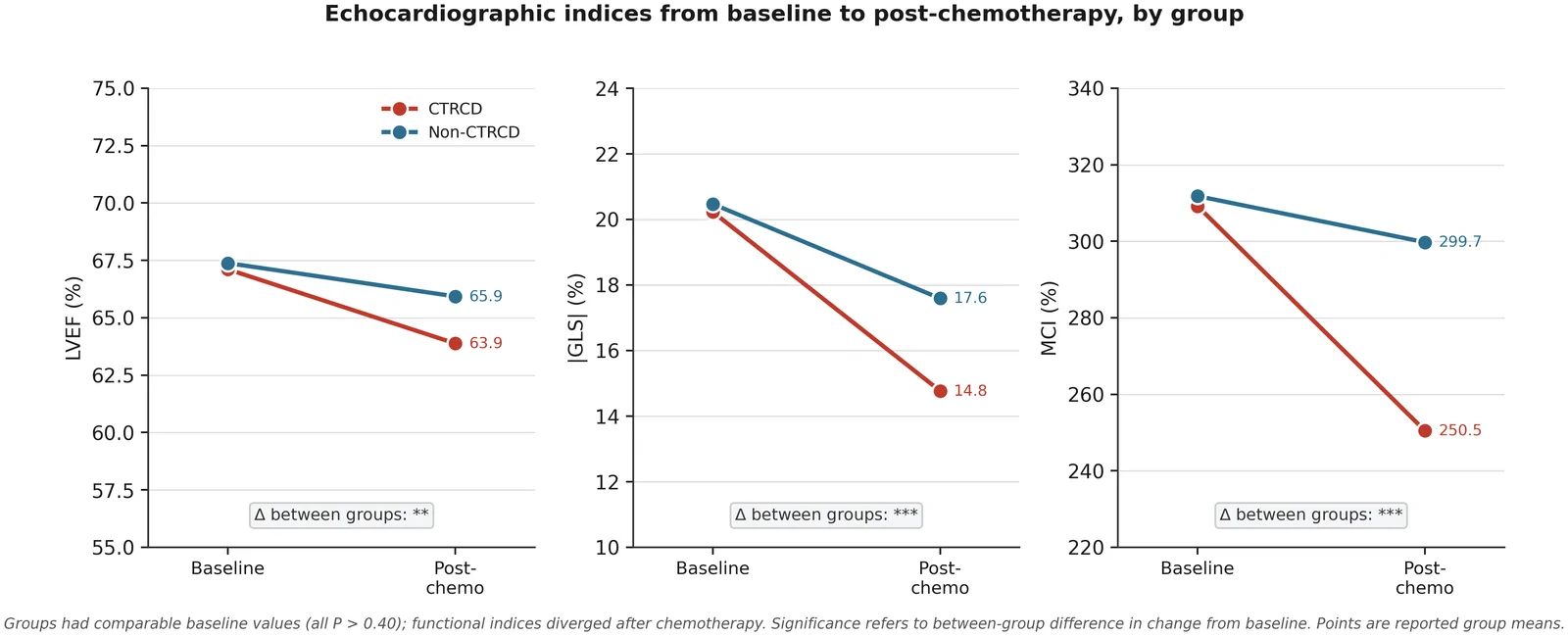
Echocardiographic indices from baseline to post-chemotherapy, by group. Left ventricular ejection fraction (LVEF), absolute global longitudinal strain (|GLS|) and myocardial composite index (MCI) were comparable at baseline (all P > 0.40) and diverged after chemotherapy; significance refers to the between-group difference in change from baseline. Points are group means.

Changes from baseline to the post-cycle-3 assessment are shown in **Table 3** and **Figure 3**. The absolute reduction in LVEF was greater in the CTRCD group than in the non-CTRCD group (−3.23 ± 4.71 vs −1.45 ± 3.82 percentage points; mean difference in change, −1.78 percentage points; 95% CI, −3.08 to −0.48; P = 0.004). The relative reduction in |GLS| was also larger in the CTRCD group (−26.4 ± 10.8% vs −13.5 ± 8.9%; mean difference in change, −12.90%; 95% CI, −15.90 to −9.90; P < 0.001). MCI decreased more substantially in the CTRCD group than in the non-CTRCD group (−58.7 ± 39.8 vs −12.1 ± 34.5; mean difference in change, −46.60; 95% CI, −57.77 to −35.43; P < 0.001). Post-cycle hs-cTnI was higher in the CTRCD group than in the non-CTRCD group (0.052 ± 0.021 vs 0.013 ± 0.009 ng/mL; P < 0.001).

**Table 3:** Baseline-to-post-treatment change in cardiac measures by group.

Measure	CTRCD (n = 66)	Non-CTRCD (n = 153)	*P* value
Baseline LVEF, %	67.11 ± 4.62	67.38 ± 4.35	0.679
Post-cycle LVEF, %	63.88 ± 5.32	65.93 ± 6.29	0.022
**Absolute LVEF change, points**	**−3.23 ± 4.71**	**−1.45 ± 3.82**	0.004
Baseline |GLS|, %	20.23 ± 2.05	20.47 ± 2.01	0.421
Post-cycle |GLS|, %	14.77 ± 2.39	17.59 ± 2.71	< 0.001
**Relative |GLS| change, %**	**−26.4 ± 10.8**	**−13.5 ± 8.9**	< 0.001
Baseline MCI, %	309.2 ± 45.6	311.8 ± 43.9	0.691
Post-cycle MCI, %	250.49 ± 41.93	299.74 ± 47.60	< 0.001
**MCI change, %**	**−58.7 ± 39.8**	**−12.1 ± 34.5**	< 0.001
Baseline hs-cTnI, ng/mL	0.008 ± 0.004	0.007 ± 0.003	0.071
**Post-cycle hs-cTnI, ng/mL**	**0.052 ± 0.021**	**0.013 ± 0.009**	< 0.001

Data are mean ± SD. Change rows (bold) are the between-group comparisons of the change from baseline. Abbreviations as in **Table 1**.

### 3.5 Correlations between serum biomarkers and echocardiographic indices

**Figure 4:**
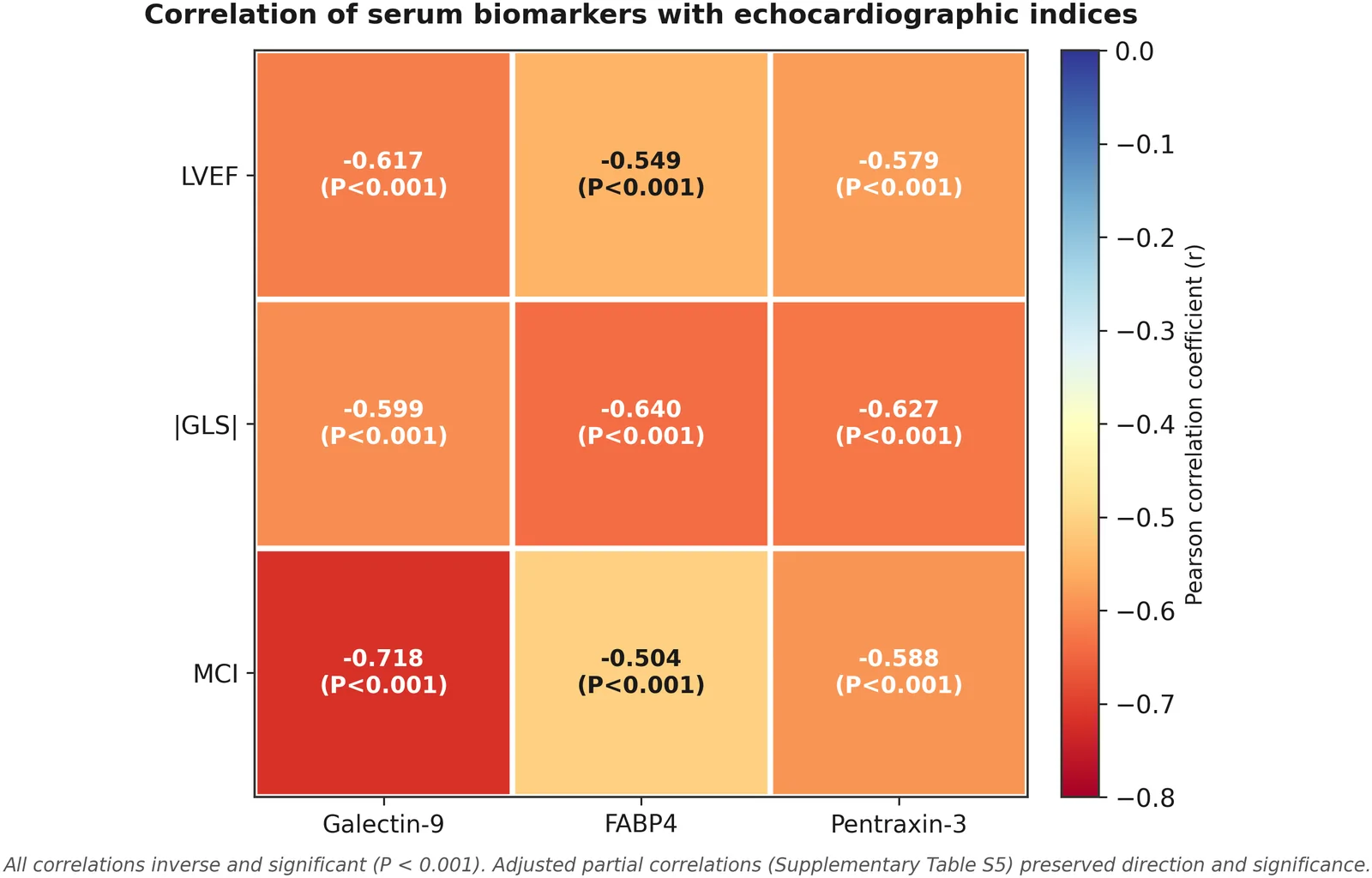
Correlation heat-map. Pearson correlation coefficients between each serum biomarker and LVEF, |GLS| and MCI; all correlations are inverse and significant (P < 0.001). Adjusted partial correlations are in **Supplementary Table 5**.

Across the full cohort, Gal-9, FABP4 and PTX-3 each showed inverse correlations with LVEF, |GLS| and MCI (**Figure 4**). Gal-9 correlated with LVEF (r = −0.617; P < 0.001), |GLS| (r = −0.599; P < 0.001) and MCI (r = −0.718; P < 0.001). FABP4 correlated with LVEF (r = −0.549; P < 0.001), |GLS| (r = −0.640; P < 0.001) and MCI (r = −0.504; P < 0.001). PTX-3 correlated with LVEF (r = −0.579; P < 0.001), |GLS| (r = −0.627; P < 0.001) and MCI (r = −0.588; P < 0.001).

After adjustment for age, body mass index, hypertension, diabetes, hyperlipidaemia, trastuzumab use, doxorubicin-equivalent anthracycline dose and baseline LVEF, the partial correlations remained inverse and statistically significant. Adjusted partial correlation coefficients ranged from −0.34 to −0.58, with all P values <0.001 (**Supplementary Table 5**).

### 3.6 Adjusted association of biomarkers with CTRCD

Multivariable logistic regression results are presented in **Table 4**. In the model including clinical covariates and the three standardised biomarkers, Gal-9 was independently associated with CTRCD (adjusted OR per SD increase, 1.59; 95% CI, 1.04–2.45; P = 0.035). PTX-3 was also independently associated with CTRCD (adjusted OR per SD increase, 1.70; 95% CI, 1.09–2.66; P = 0.019). FABP4 showed a positive association that did not reach conventional statistical significance (adjusted OR per SD increase, 1.42; 95% CI, 0.96–2.13; P = 0.080).

**Table 4:** Multivariable logistic regression for cancer therapy-related cardiac dysfunction.

Variable	Adjusted OR	95% CI	*P*
Model A — clinical covariates and individual biomarkers			
Age, per 5 years	1.10	0.76–1.59	0.614
Body mass index, per 1 kg/m^2^	1.12	0.76–1.66	0.568
Hypertension	1.19	0.52–2.72	0.681
Diabetes	1.08	0.41–2.86	0.876
Hyperlipidaemia	1.24	0.52–2.93	0.627
Trastuzumab use	1.51	0.90–2.74	0.117
Doxorubicin-equivalent dose, per 50 mg/m^2^	1.21	0.88–1.67	0.232
Baseline LVEF, per SD	0.91	0.63–1.33	0.630
**Gal-9, per SD**	**1.59**	1.04–2.45	**0.035**
FABP4, per SD	1.42	0.96–2.13	0.080
**PTX-3, per SD**	**1.70**	1.09–2.66	**0.019**
Model B — combined biomarker score with key confounders			
**Combined Gal-9 + FABP4 + PTX-3 score, per SD**	**3.25**	2.09–5.07	**< 0.001**
Trastuzumab use	1.43	0.81–2.53	0.214
Doxorubicin-equivalent dose, per 50 mg/m^2^	1.18	0.86–1.61	0.306
Baseline LVEF, per SD	0.94	0.66–1.35	0.747

Biomarkers standardised (odds ratio per 1-SD increase). The combined score is the logistic-regression-derived predicted probability of the three biomarkers. CI, confidence interval; OR, odds ratio; other abbreviations as in **Table 1**.

Bold values indicate statistically significant biomarker associations (P < 0.05).

In the model using the combined Gal-9, FABP4 and PTX-3 score, the combined biomarker score was associated with higher odds of CTRCD after adjustment for trastuzumab use, doxorubicin-equivalent dose and baseline LVEF (adjusted OR per SD increase, 3.25; 95% CI, 2.09–5.07; P < 0.001). Trastuzumab use, doxorubicin-equivalent dose and baseline LVEF were not independently associated with CTRCD in this model.

### 3.7 Discrimination of CTRCD and internal validation

**Figure 5:**
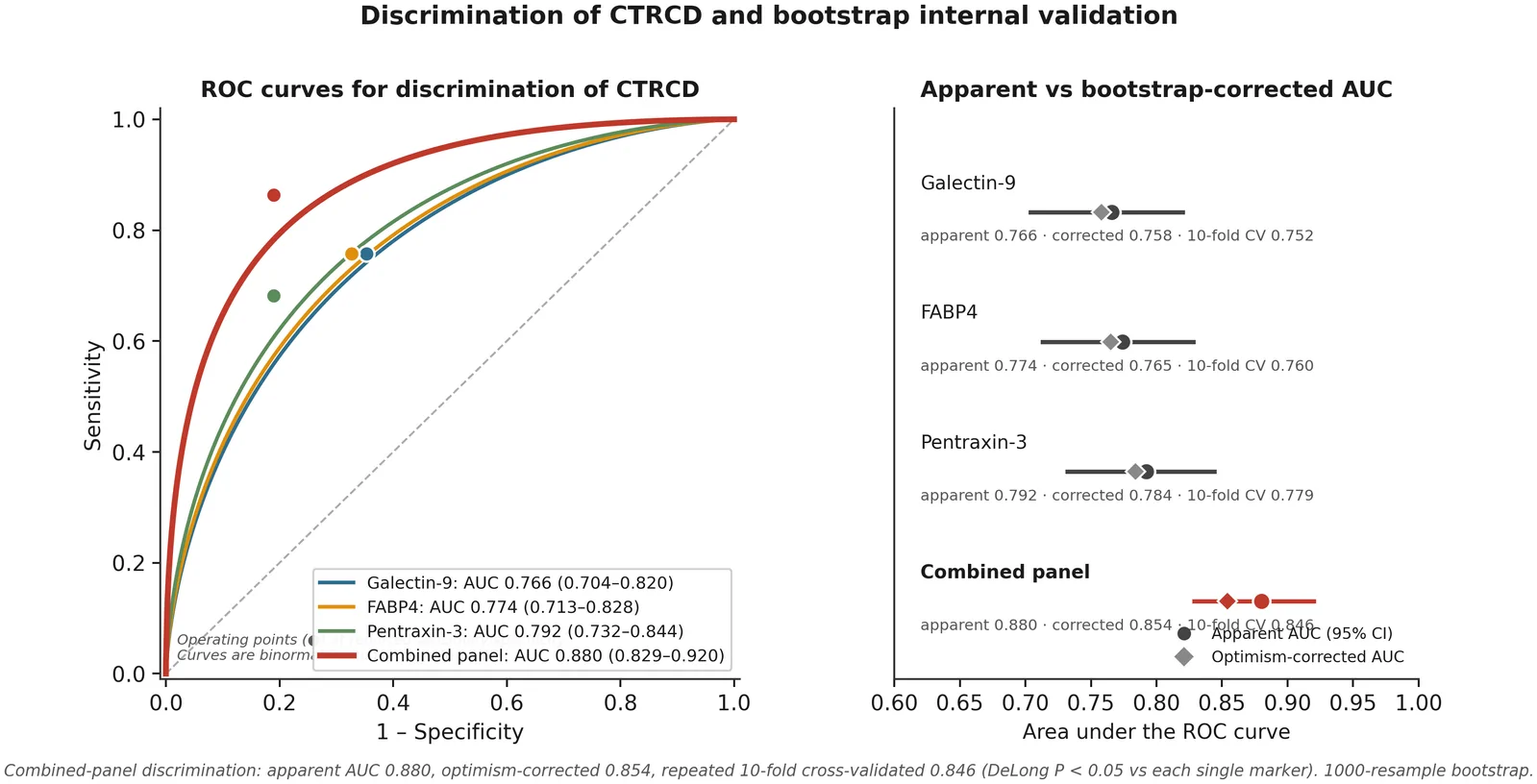
Discrimination of CTRCD and internal validation. Left, ROC curves for each biomarker and the combined model, with operating points (circles) at the reported sensitivity/specificity; curves are binormal fits to the reported AUCs. Right, apparent AUC (95% CI) and bootstrap optimism-corrected AUC. The combined model significantly outperformed each single marker (DeLong P < 0.05).

The discriminatory performance of individual biomarkers and the combined biomarker model is shown in **Table 5** and **Figure 5**. Gal-9 had an apparent AUC of 0.766 (95% CI, 0.704–0.820), with sensitivity of 75.8% and specificity of 64.7% at a cut-off of 7.36 ng/mL. FABP4 had an apparent AUC of 0.774 (95% CI, 0.713–0.828), with sensitivity of 75.8% and specificity of 67.3% at a cut-off of 39.06 ng/mL. PTX-3 had an apparent AUC of 0.792 (95% CI, 0.732–0.844), with sensitivity of 68.2% and specificity of 81.1% at a cut-off of 5.93 ng/mL.

**Table 5:** Discrimination of CTRCD and bootstrap internal validation.

Marker	Cut-off	AUC	95% CI	Sens %	Spec %	Corrected AUC	10-fold CV AUC
Gal-9 (ng/mL)	7.36	0.766	0.704–0.820	75.8	64.7	0.758	0.752
FABP4 (ng/mL)	39.06	0.774	0.713–0.828	75.8	67.3	0.765	0.760
PTX-3 (ng/mL)	5.93	0.792	0.732–0.844	68.2	81.1	0.784	0.779
**Combined panel**	—	**0.880**	0.829–0.920	86.4	81.1	0.854	0.846

AUC, apparent area under the ROC curve; corrected AUC, 1000-resample bootstrap optimism-corrected AUC; CV, cross-validated. The combined model outperformed each single marker (DeLong P < 0.05) and showed a calibration slope of 0.86 and a Brier score of 0.134. CI, confidence interval; Sens, sensitivity; Spec, specificity.

Bold values indicate the combined biomarker panel, which showed the highest discrimination.

The combined biomarker panel had an apparent AUC of 0.880 (95% CI, 0.829–0.920), with sensitivity of 86.4% and specificity of 81.1%. The combined model outperformed each individual biomarker on DeLong comparison (P < 0.05 for each comparison). In bootstrap internal validation, the optimism-corrected AUC for the combined model was 0.854. The repeated 10-fold cross-validated AUC was 0.846. The calibration slope was 0.86 and the Brier score was 0.134.

### 3.8 Incremental discrimination beyond clinical variables and decision-curve analysis

Incremental discrimination beyond standard clinical information is summarised in **Table 6**. The clinical model, which included age, body mass index, hypertension, diabetes, hyperlipidaemia, trastuzumab use, doxorubicin-equivalent dose and baseline LVEF, had an AUC of 0.642 (95% CI, 0.570–0.710). Adding Gal-9 increased the AUC to 0.789 (95% CI, 0.728–0.842; ΔAUC, 0.147; P < 0.001). Adding FABP4 increased the AUC to 0.800 (95% CI, 0.740–0.852; ΔAUC, 0.158; P < 0.001), and adding PTX-3 increased the AUC to 0.811 (95% CI, 0.751–0.861; ΔAUC, 0.169; P < 0.001).

**Table 6:** Incremental discrimination and reclassification beyond the clinical model.

Model	AUC	95% CI	ΔAUC	*P*	IDI/NRI
Clinical model only	0.642	0.570–0.710	Ref.	—	—
Clinical + Gal-9	0.789	0.728–0.842	0.147	< 0.001	0.096/0.38
Clinical + FABP4	0.800	0.740–0.852	0.158	< 0.001	0.104/0.41
Clinical + PTX-3	0.811	0.751–0.861	0.169	< 0.001	0.116/0.44
**Clinical + all three biomarkers**	**0.872**	0.818–0.914	0.230	< 0.001	0.184/0.57

Clinical model: age, body mass index, hypertension, diabetes, hyperlipidaemia, trastuzumab use, doxorubicin-equivalent dose and baseline LVEF. ΔAUC and P are versus the clinical model (DeLong). IDI, integrated discrimination improvement; NRI, category-free net reclassification improvement.

Bold values indicate the clinical model plus all three biomarkers, which showed the greatest incremental discrimination.

**Figure 6:**
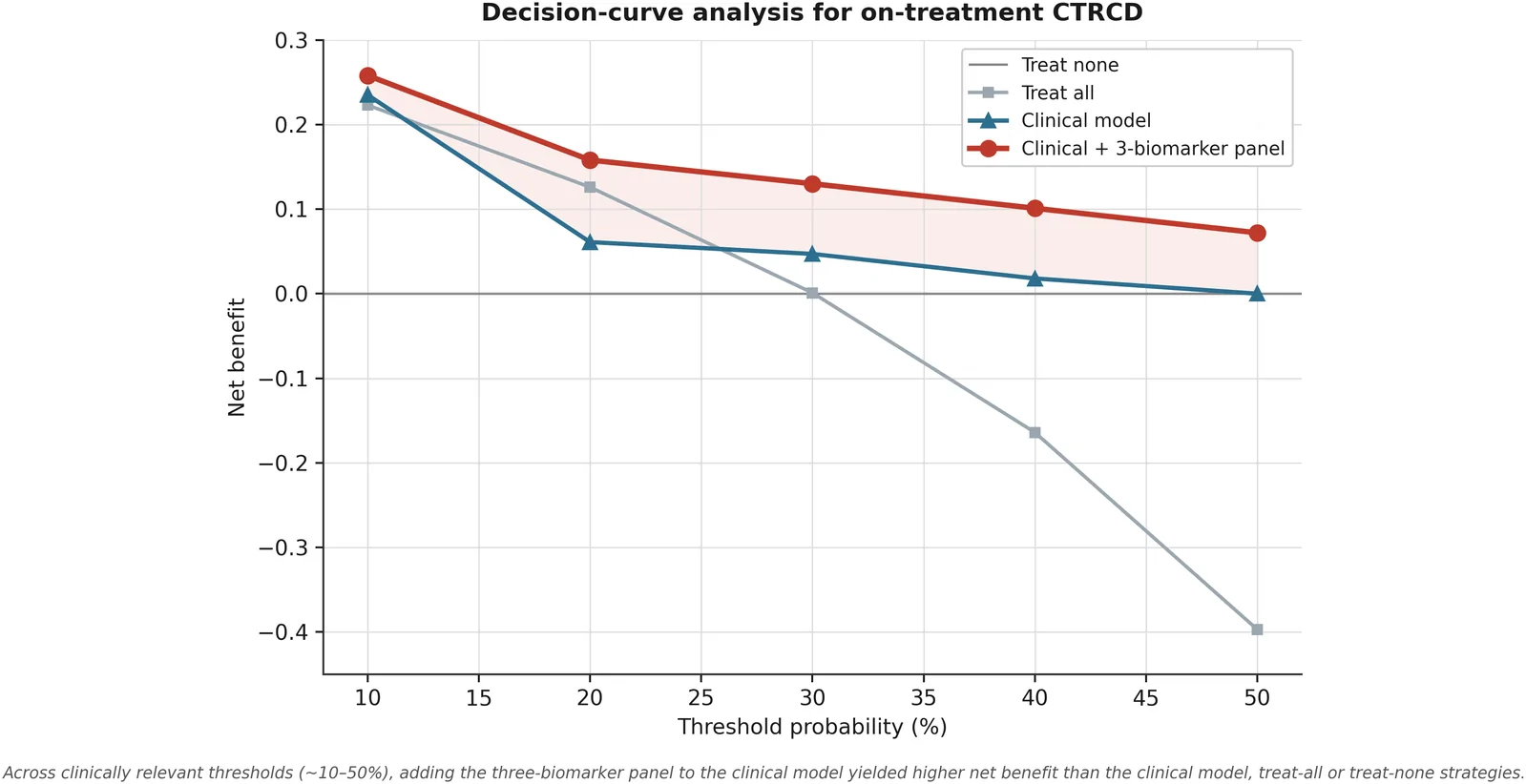
Decision-curve analysis for on-treatment CTRCD. Across threshold probabilities of roughly 10–50%, adding the three-biomarker panel to the clinical model yielded higher net benefit than the clinical model, treat-all or treat-none strategies.

The addition of all three biomarkers to the clinical model produced the highest discrimination, with an AUC of 0.872 (95% CI, 0.818–0.914), representing an increase of 0.230 compared with the clinical model alone (P < 0.001). The corresponding integrated discrimination improvement was 0.184 and the category-free net reclassification improvement was 0.57. Decision-curve analysis showed that the clinical model plus the three-biomarker panel had higher net benefit than the clinical model alone, treat-all strategy or treat-none strategy across threshold probabilities of approximately 10–50% (**Figure 6**).

### 3.9 Sensitivity s

Pre-specified sensitivity analyses are reported in **Supplementary Table 6**. After excluding trastuzumab-treated patients, the combined biomarker panel had an AUC of 0.861 (95% CI, 0.794–0.914) among 160 patients with 43 CTRCD events. In the epirubicin-only subgroup, the AUC was 0.872 (95% CI, 0.814–0.918) among 180 patients with 52 events. When the outcome was restricted to imaging-supported CTRCD based on LVEF and/or |GLS| criteria, the AUC was 0.858 (95% CI, 0.795–0.907) among 202 patients with 49 events. After excluding patients classified as CTRCD solely on the basis of hs-cTnI elevation, the AUC was 0.835 (95% CI, 0.742–0.903) among 177 patients with 24 events. In the analysis adjusted for clinical covariates, the AUC was 0.867 (95% CI, 0.812–0.910). After additional adjustment for tumour stage and tumour size, the AUC was 0.865, indicating that the combined-panel discrimination was materially unchanged.

### 3.10 Safety and treatment-related events

No patient was excluded because of a treatment modification prompted by a cardiac adverse event. The regimen modifications observed during the study period were attributed to non-cardiac toxicities. New symptomatic heart failure occurred in 4 of 66 patients with CTRCD (6.1%), and intervention-requiring arrhythmia occurred in 8 of 66 patients with CTRCD (12.1%), as shown in **Supplementary Table 1**. No additional safety outcomes, treatment-related mortality or readmission data were available for analysis in the current dataset.

## 4 Discussion

In this single-centre retrospective cohort of women with breast cancer receiving anthracycline-based chemotherapy, serum Gal-9, FABP4 and PTX-3 were each associated with on-treatment CTRCD and with concurrent echocardiographic evidence of myocardial dysfunction. The biomarkers were higher in patients with CTRCD, correlated inversely with LVEF, |GLS| and MCI, and retained significant associations after adjustment for relevant clinical and treatment-related factors. When combined, the three-marker panel discriminated CTRCD more strongly than any individual biomarker, remained stable after bootstrap optimism correction and cross-validation, and improved discrimination beyond a clinical model. These findings suggest that a biologically complementary panel reflecting immune-inflammatory activation, lipid-metabolic stress and acute-phase vascular injury may capture on-treatment myocardial stress more comprehensively than a single circulating marker. Because biomarker sampling and outcome assessment were performed within the same treatment window, the results should be interpreted as associative and discriminatory rather than predictive.

An important interpretive issue is the definition of CTRCD. In this study, CTRCD was adjudicated using imaging, strain, troponin and clinical criteria consistent with contemporary cardio-oncology practice. Because LVEF decline contributed to group classification, the lower post-treatment LVEF in the CTRCD group should be viewed as confirmatory rather than fully independent evidence. The more informative findings are the concordant deterioration in |GLS| and MCI, the greater baseline-to-post-treatment change in these indices, and the persistence of the biomarker signal in sensitivity analyses restricted to imaging-supported CTRCD or excluding hs-cTnI-only cases. These analyses reduce the concern that the biomarker findings simply reflected the way cases were defined. This point deserves particular emphasis because hs-cTnI is itself a circulating biomarker embedded within the composite endpoint, which raises a legitimate concern about circularity when a biomarker-inclusive outcome is used to evaluate a new biomarker panel. Two features of the design mitigate this concern. First, the candidate analytes are biochemically independent of troponin and did not enter the CTRCD definition. Second, the panel retained strong discrimination when the outcome was restricted to imaging-supported CTRCD defined by LVEF and |GLS| criteria (AUC 0.858) and when troponin-only cases were removed (AUC 0.835), indicating that the signal is not an artefact of the troponin component of the definition.

The imaging results are consistent with current understanding of chemotherapy-related myocardial injury. LVEF is clinically familiar but may remain preserved until myocardial reserve is substantially reduced. GLS reflects longitudinal deformation and is more sensitive to early subclinical systolic dysfunction, particularly in patients exposed to cardiotoxic cancer therapy (7, 8, 17). In the present cohort, |GLS| and MCI showed clearer separation between groups than LVEF, whereas radial strain and diastolic indices did not differ significantly. This pattern is compatible with earlier impairment of longitudinal deformation compared with conventional global pump function (17, 18). It also aligns with the vulnerability of subendocardial longitudinal fibres during anthracycline exposure (7). The parallel decline in LVEF, |GLS| and MCI supports the role of multiparametric echocardiographic assessment when evaluating myocardial effects of breast cancer therapy (6, 19, 20).

The biomarker findings are biologically plausible, although this study was not designed to prove mechanism. Gal-9 participates in immune regulation and inflammatory signalling and may contribute to myocardial remodelling through interaction with T-cell immunoglobulin and mucin-domain 3, with downstream activation of cytokine networks relevant to myocardial stress and contractile dysfunction (10, 21). FABP4 links adipose-tissue metabolism, fatty-acid handling and inflammatory activation; in chemotherapy-related myocardial stress, elevated FABP4 may reflect altered lipid mobilisation, lipotoxicity, mitochondrial dysfunction or impaired energetic adaptation (9). PTX-3 is released during vascular and myocardial inflammation and has been linked to complement activation, endothelial injury and adverse cardiovascular outcomes (11, 22). Anthracycline-associated injury involves oxidative stress, mitochondrial damage, inflammation, endothelial dysfunction and remodelling; therefore, a panel spanning several biological axes may show stronger discrimination than one marker alone.

The combined-panel result is clinically relevant because CTRCD is heterogeneous. Some patients develop measurable ventricular dysfunction, some show strain abnormalities, and others present mainly with cardiac biomarker elevation. A single analyte is unlikely to capture this heterogeneity fully. In this study, each biomarker had moderate individual discriminatory performance, whereas the combined score showed stronger discrimination and retained acceptable performance after internal validation. The three markers were moderately intercorrelated, which is expected given their overlapping involvement in the inflammatory and metabolic responses to myocardial stress. This modest collinearity, reflected in pairwise correlations of approximately 0.49 to 0.57 and variance inflation factors below 2.5 (**Supplementary Table 7**), is the most plausible explanation for the wider confidence interval and the non-significant independent P value for FABP4 in the fully adjusted model, even though FABP4 showed a clear univariable association with CTRCD and contributed materially to the combined logistic score. Collinearity of this degree inflates standard errors and can suppress the apparent independence of individual predictors without diminishing their joint contribution, which is precisely the pattern observed here and the reason the combined score, rather than any single marker, is proposed as the unit of interest. The improvement over the clinical model is important because standard demographic, cardiovascular and treatment variables alone only weakly separated patients with and without CTRCD. Decision-curve analysis further suggested that adding the biomarker panel may provide higher net benefit across clinically plausible thresholds. These findings do not justify immediate clinical implementation, but they support further evaluation of Gal-9, FABP4 and PTX-3 as candidate adjunctive biomarkers for cardio-oncology surveillance.

The present results extend prior work rather than duplicate it. Previous studies have evaluated troponin, natriuretic peptides and other emerging biomarkers in patients receiving anthracycline-based breast cancer therapy (9, 23). A recent report also examined galectin and pentraxin markers in anthracycline-treated breast cancer (24). The current study adds to this literature by incorporating FABP4 as a lipid-metabolic stress marker, relating the three biomarkers to multiple echocardiographic functional indices rather than to LVEF alone, and evaluating the combined panel using multivariable adjustment, internal validation, incremental-discrimination analysis and decision-curve analysis. These elements strengthen the clinical and statistical assessment of the biomarker signal, although external validation remains essential.

A further consideration is whether the biomarker differences reflect chemotherapy-related myocardial injury specifically or the underlying malignancy itself, because each of these analytes can be influenced by tumour biology. Serum galectin-9 has been reported to rise in HER2-enriched breast cancer (25), circulating FABP4 is elevated chiefly in association with adiposity and has been linked to breast tumour progression (26), and pentraxin-3 increases with tumour grade and inflammatory burden in breast cancer (27). Several features of the present design reduce the likelihood that the observed signal is driven by the cancer process rather than by cardiac injury. Baseline tumour characteristics, including stage, histological subtype, tumour size and HER2 status, together with body mass index, were closely balanced between the CTRCD and non-CTRCD groups (**Table 1**), so differential tumour burden or adiposity is unlikely to explain the between-group biomarker differences that emerged only after chemotherapy. In addition, the biomarkers correlated with concurrent deterioration of cardiac function, including |GLS| and MCI, after adjustment for treatment exposure and baseline risk (**Supplementary Table 5**); a signal generated solely by tumour biology would not be expected to track cardiac functional decline in this way. To probe this further, we repeated the combined-panel analysis with additional adjustment for tumour stage and tumour size, and discrimination was essentially unchanged (AUC 0.865; **Supplementary Table 6**). We nonetheless acknowledge that a retrospective design cannot fully separate the cardiac from the oncological contributions to these markers, a point that is considered further in the study limitations and in our proposals for future work.

Several practical implications can be drawn cautiously. In routine cardio-oncology care, surveillance commonly relies on echocardiography, LVEF, GLS and established biomarkers such as troponin or natriuretic peptides. The present findings suggest that Gal-9, FABP4 and PTX-3 may provide additional information about concurrent myocardial stress during anthracycline therapy. If validated prospectively, such a panel could help identify patients who warrant closer imaging surveillance, cardiology referral, intensified cardiovascular risk management or consideration of cardioprotective strategies. The panel should be considered an adjunct to imaging and established cardiac biomarkers, not a replacement for them. Because this was a retrospective study with single-time-point biomarker sampling, the findings should not be interpreted as defining a screening threshold, treatment algorithm or stand-alone diagnostic test.

### 4.1 Strengths and limitations

This study has several strengths. It included consecutive women treated in a real-world oncology setting during a defined period, with clearly specified eligibility criteria, treatment exposure and outcome definition. Baseline cardiac function was documented, allowing evaluation of on-treatment change rather than reliance on post-treatment values alone. Biomarker assays and echocardiography were performed within a defined assessment window, and personnel were blinded to clinical grouping where relevant. The analysis incorporated covariate-adjusted correlations, multivariable logistic regression, internal validation, incremental-discrimination analysis and pre-specified sensitivity analyses. These sensitivity analyses addressed potential concerns related to trastuzumab exposure, anthracycline type, troponin-defined CTRCD and imaging-supported outcome definitions. The use of a uniform post-treatment sampling window, common sample-processing procedures, single-batch biomarker assays and a consistent echocardiographic platform further strengthened internal measurement consistency, while not resolving the need for evaluation in other clinical settings.

Several limitations should be acknowledged. First, the single-centre retrospective design limits generalisability and remains vulnerable to selection bias, information bias and residual confounding. Second, biomarkers were measured at one on-treatment time point concurrent with CTRCD ascertainment; therefore, the study cannot determine whether biomarker elevation preceded functional decline. Third, although bootstrap correction and cross-validation were performed, external validation in an independent cohort was unavailable. These internal procedures quantify apparent-sample optimism and reproducibility within the present dataset, but they cannot establish transportability across institutions, patient populations, assay platforms or imaging systems. Fourth, some CTRCD cases were defined by hs-cTnI elevation, and troponin-defined and imaging-defined phenotypes may not be biologically identical, although sensitivity analyses partly addressed this issue. Fifth, MCI was vendor-derived and should be considered exploratory. Finally, the study did not include serial biomarker trajectories, mechanistic experiments or long-term cardiovascular outcomes, and therefore cannot address delayed cardiotoxicity or causal biological pathways. As a single-centre study, the findings require external, multicentre confirmation before any firm inference about generalisability can be drawn, and the panel is therefore presented as a candidate requiring prospective multicentre validation rather than as a validated clinical tool. The exploratory myocardial composite index was derived from proprietary Philips software, and because deformation and vendor-specific indices differ across ultrasound platforms (28, 29), the MCI values reported here should not be transferred directly to other vendors or used to define cross-platform thresholds. In addition, and as discussed above, the three biomarkers can be influenced by tumour biology; although baseline tumour characteristics were balanced and additional adjustment for tumour stage and size did not alter discrimination, residual confounding by the malignancy itself cannot be entirely excluded.

Future research should evaluate the biomarker panel prospectively in a multicentre cohort using harmonised eligibility criteria, biomarker sampling and laboratory procedures, standardised CTRCD adjudication, and echocardiographic assessment across multiple ultrasound vendors. Such evaluation should examine discrimination, calibration and clinical usefulness across participating institutions, treatment protocols, assay platforms and imaging systems.

### 4.2 Conclusions

In conclusion, serum Gal-9, FABP4 and PTX-3 were associated with concurrent echocardiographic myocardial dysfunction in women receiving anthracycline-based chemotherapy for breast cancer. A combined three-biomarker panel discriminated on-treatment CTRCD better than individual markers and added value beyond standard clinical information, with performance that remained acceptable after internal validation and sensitivity analyses. These findings provide preliminary evidence that biologically complementary biomarkers may help characterize on-treatment myocardial stress in breast cancer patients receiving anthracyclines. Prospective serial sampling, external validation and longer-term outcome studies are required before this panel can be considered for routine clinical use.

## Data Availability

The original contributions presented in the study are included in the article/**Supplementary Material**. Further inquiries can be directed to the corresponding author.
