# Supplementary material for: Galectin-9, FABP4 and pentraxin-3 are associated with myocardial dysfunction and discriminate chemotherapy-related cardiac dysfunction in anthracycline-treated breast cancer: a single-centre retrospective study
Source: Front Oncol. 2026 Aug 26;16:1913970. doi: 10.3389/fonc.2026.1913970 (PMC13553379; doi:10.3389/fonc.2026.1913970)
Supplement: Supplementary file 1 [file DataSheet1.docx]

**Supplementary Material**

**Supplementary Table S1. Components of the CTRCD diagnosis**

Diagnostic criteria were assessed per the 2014 ASE/EACVI consensus and 2022 ESC cardio-oncology guideline. Because criteria overlap, both the non-mutually-exclusive component frequencies and a mutually exclusive primary classification are shown.

| **CTRCD component (non-mutually exclusive)** | **n / 66** | **%** |
| --- | --- | --- |
| LVEF reduction > 10 points to < 53% | 10/66 | 15.2 |
| hs-cTnI above 99th-percentile URL | 51/66 | 77.3 |
| New relative \|GLS\| decline > 15% from baseline | 49/66 | 74.2 |
| New symptomatic heart failure | 4/66 | 6.1 |
| Intervention-requiring arrhythmia | 8/66 | 12.1 |
| More than one primary criterion | 9/66 | 13.6 |

Mutually exclusive primary classification:

| **Primary classification pathway** | **n** | **%** |
| --- | --- | --- |
| hs-cTnI elevation only | 42 | 63.6 |
| LVEF criterion only | 5 | 7.6 |
| Heart-failure symptoms only | 2 | 3.0 |
| Intervention-requiring arrhythmia only | 8 | 12.1 |
| hs-cTnI + LVEF criterion | 5 | 7.6 |
| hs-cTnI + heart failure / arrhythmia | 4 | 6.1 |
| Total CTRCD | 66 | 100 |

hs-cTnI, high-sensitivity cardiac troponin I; LVEF, left ventricular ejection fraction; |GLS|, absolute global longitudinal strain; URL, upper reference limit. Sensitivity analyses excluding hs-cTnI-only cases and restricting to imaging-supported CTRCD are reported in Supplementary Table S6.

**Supplementary Table S2. ELISA assay characteristics**

| **Analyte** | **Manufacturer / catalogue** | **Detection range** | **LOD** | **Intra-/inter-assay CV** | **Storage** | **Batch handling** |
| --- | --- | --- | --- | --- | --- | --- |
| Gal-9 | Wuhan Fine Biotech Co., Ltd. / FineTest, EH0148 | 0.156–10 ng/mL | 0.094 ng/mL | < 10% / < 15% | −80 °C | Single batch; blinded |
| FABP4 | Elabscience Biotechnology Inc., E-EL-H0285] | 0.39–25 ng/mL | 0.23 ng/mL | < 10% / < 15% | −80 °C | Single batch; blinded |
| PTX-3 | Elabscience Biotechnology Inc., E-EL-H6081 | 6.86–5000 pg/mL | 4.12 pg/mL | < 10% / < 15% | −80 °C | Single batch; blinded |

CV, coefficient of variation; LOD, lower limit of detection.

**Supplementary Table S3. Timing of biomarker and echocardiographic assessments**

| **Timing variable** | **CTRCD (n=66)** | **Non-CTRCD (n=153)** | **P** |
| --- | --- | --- | --- |
| Baseline echocardiography before chemotherapy, days | 6.0 (4.0–9.0) | 6.0 (4.0–8.0) | 0.684 |
| Baseline hs-cTnI before chemotherapy, days | 5.0 (3.0–8.0) | 5.0 (3.0–8.0) | 0.912 |
| Blood sampling after third cycle, hours | 18.0 (12.0–22.0) | 17.0 (12.0–23.0) | 0.771 |
| Echocardiography after third cycle, hours | 32.0 (24.0–46.0) | 30.0 (24.0–44.0) | 0.508 |
| Blood-to-echo interval, hours | 11.0 (6.0–20.0) | 10.0 (5.0–19.0) | 0.642 |
| Patients with blood and echo within 48 h, n (%) | 61 (92.4) | 143 (93.5) | 0.772 |

Data are median (interquartile range) unless stated. Timing did not differ between groups, indicating comparable assessment windows.

**Supplementary Table S4. Echocardiographic reproducibility**

| **Parameter** | **Intraobserver ICC** | **Interobserver ICC** | **Intraobserver CV %** | **Interobserver CV %** |
| --- | --- | --- | --- | --- |
| LVEF | 0.93 | 0.90 | 4.8 | 6.1 |
| E/e′ | 0.88 | 0.84 | 6.5 | 8.2 |
| E/A | 0.90 | 0.87 | 5.9 | 7.4 |
| GRS | 0.89 | 0.85 | 7.1 | 8.8 |
| \|GLS\| | 0.95 | 0.92 | 4.1 | 5.3 |
| MCI | 0.91 | 0.88 | 5.8 | 7.0 |

ICC, intraclass correlation coefficient; CV, coefficient of variation. Reproducibility was good to excellent for all indices, including the exploratory MCI.

**Supplementary Table S5. Partial correlations between biomarkers and echocardiographic indices**

Adjusted for age, body mass index, hypertension, diabetes, hyperlipidaemia, trastuzumab use, doxorubicin-equivalent anthracycline dose and baseline LVEF.

| **Index** | **Gal-9** | | **FABP4** | | **PTX-3** | |
| --- | --- | --- | --- | --- | --- | --- |
|  | ***r*** | ***P*** | ***r*** | ***P*** | ***r*** | ***P*** |
| LVEF | −0.46 | < 0.001 | −0.37 | < 0.001 | −0.43 | < 0.001 |
| \|GLS\| | −0.48 | < 0.001 | −0.52 | < 0.001 | −0.51 | < 0.001 |
| MCI | −0.58 | < 0.001 | −0.34 | < 0.001 | −0.46 | < 0.001 |

All partial correlations remained inverse and significant after adjustment, indicating biomarker–function associations independent of treatment exposure and baseline risk. Abbreviations as in the main text.

**Supplementary Table S6. Sensitivity analyses for combined-panel discrimination**

| **Analysis** | **N** | **Events** | **AUC** | **95% CI** |
| --- | --- | --- | --- | --- |
| Primary analysis | 219 | 66 | 0.880 | 0.829–0.920 |
| Bootstrap optimism-corrected | 219 | 66 | 0.854 | — |
| Excluding trastuzumab-treated patients | 160 | 43 | 0.861 | 0.794–0.914 |
| Epirubicin-only subgroup | 180 | 52 | 0.872 | 0.814–0.918 |
| Imaging-supported CTRCD only | 202 | 49 | 0.858 | 0.795–0.907 |
| Excluding hs-cTnI-only CTRCD cases | 177 | 24 | 0.835 | 0.742–0.903 |
| Adjusted for clinical covariates | 219 | 66 | 0.867 | 0.812–0.910 |
| Additionally adjusted for tumour stage and size | 219 | 66 | 0.865 | 0.808–0.909 |

Imaging-supported CTRCD: cases meeting the LVEF and/or > 15% |GLS|-decline criteria. Discrimination remained in the acceptable range across all analyses, supporting robustness to outcome definition, anthracycline type and trastuzumab exposure. Additional adjustment for tumour stage and tumour size left discrimination essentially unchanged, indicating that the biomarker signal is not explained by differential tumour burden. AUC, area under the ROC curve; CI, confidence interval.

**Supplementary Table S7. Collinearity diagnostics for the three biomarkers**

Pairwise Pearson correlation coefficients among the three biomarkers and variance inflation factors (VIF) from the multivariable logistic-regression model. Correlations were of moderate magnitude and all VIF values were below 2.5.

| **Biomarker** | **r with Gal-9** | **r with FABP4** | **r with PTX-3** | **VIF** |
| --- | --- | --- | --- | --- |
| Gal-9 | — | 0.52 | 0.57 | 2.18 |
| FABP4 | 0.52 | — | 0.49 | 1.92 |
| PTX-3 | 0.57 | 0.49 | — | 2.27 |

VIF, variance inflation factor. A VIF below 5 is conventionally regarded as acceptable and below 2.5 as reflecting only mild-to-moderate collinearity; the mean VIF here was 2.12. Moderate correlation among the markers is expected given their overlapping inflammatory and metabolic roles and is the most likely reason for the wider confidence interval and non-significant independent P value of FABP4 in the fully adjusted model, without diminishing its contribution to the combined score.

**Supplementary Note S1. STROBE reporting**

This observational cohort study is reported in accordance with the STROBE statement. A completed STROBE checklist mapping each item to the relevant manuscript section is available from the corresponding author and can be supplied as a separate file at submission. Key design elements—single-centre retrospective cohort, eligibility criteria, outcome definition (2014 ASE/EACVI; 2022 ESC), blinded assessment and the pre-specified statistical plan—are described in Section 2 of the main text; the cohort flow with exact exclusion counts is shown in Figure 1.
